# Assessment of Bacterial Diversity and Rhizospheric Community Shifts in Maize (*Zea mays* L.) Grown in Soils with Contrasting Productivity Levels

**DOI:** 10.3390/plants15010130

**Published:** 2026-01-02

**Authors:** Sebastian Cano-Serrano, Hugo G. Castelán-Sánchez, Helen Oyaregui-Cabrera, Luis G. Hernández, Ma. Cristina Pérez-Pérez, Gustavo Santoyo, Ma. del Carmen Orozco-Mosqueda

**Affiliations:** 1 Department of Biochemical and Environmental Engineering, National Technological Institute of Mexico in Celaya, Celaya 38010, Guanajuato, Mexico; m2203061@itcelaya.edu.mx (S.C.-S.); 20031765@itcelaya.edu.mx (H.O.-C.); 20031152@itcelaya.edu.mx (L.G.H.); cristina.perez@itcelaya.edu.mx (M.C.P.-P.); 2 Department of Pathology and Laboratory Medicine, University of Western Ontario, London, ON N6A 3K7, Canada; hcastelans@gmail.com; 3 Institute of Chemical-Biological Research, Universidad Michoacana de San Nicolás de Hidalgo, Morelia 58030, Michoacán, Mexico

**Keywords:** PGPR, sustainable agriculture, ASVs, plant-microbiome interactions

## Abstract

The resident microbiota in agricultural soils strongly influences crop health and productivity. In this study, we evaluated the prokaryotic diversity of two clay soils with similar physicochemical characteristics but contrasting levels of maize (*Zea mays* L.) and wheat (*Triticum aestivum* L.) production using 16S rRNA gene sequencing. Yield records showed significant differences in grain production over five consecutive years. When comparing prokaryotic alpha diversity between the “non-productive” and “productive” soils, no major differences were found, and the abundance of ammonia-oxidizing archaea (AOA) and bacterial genera such as *Arthrobacter*, *Neobacillus*, and *Microvirga* remained consistent across soils. Analysis of the top 20 genera showing the greatest abundance shifts by compartment (bulk soil vs. rhizosphere) revealed that genera such as *Priestia*, *Neobacillus*, *Sporosarcina*, and *Pontibacter* decreased in the rhizosphere of the non-productive soil, while in the productive soil, these genera remained unchanged. In the non-productive soil, genera such as *Flavisobacter* decreased in abundance in the rhizosphere, whereas *Arthrobacter* increased. Principal coordinates analysis (PCoA) showed no clear clustering by compartment (bulk vs. rhizosphere), but two distinct clusters emerged when grouping by soil type (productive vs. non-productive). Interaction networks varied by soil type: non-productive soils showed positive *Candidatus*–*Bacillus* and negative *Massilia* links, while productive soils were dominated by *Flavisolibacter* and negative *Pontibacter*. Across soils, *Rhizobium*–*Bradyrhizobium* associations were positive, whereas *Neobacillus* and *Priestia* were negative. These findings highlight that a few potential beneficial microbiota and their interactions may be key drivers of soil productivity, representing targets for microbiome-based agricultural management.

## 1. Introduction

Given the constant growth of the global human population and the need to feed it, we have placed ourselves under a continuous imperative to understand and improve agricultural production systems [1]. Since the Green Revolution, which began in the 1940s in countries such as Mexico, the use of agrochemicals—including chemical fertilizers, pesticides, and herbicides—has increased dramatically to boost crop yields. In many cases, efforts focused on improving existing crop varieties, such as wheat and maize, in which Dr. Norman Borlaug played a central role through the development of high-yielding, disease-resistant wheat varieties. While chemical fertilizers remain essential for the rapid and reliable supply of macronutrients required in intensive agricultural systems, bioinoculants have emerged as complementary tools that enhance nutrient use efficiency, support plant health, and contribute to more sustainable production practices rather than fully replacing conventional inputs [2]. Starting in the 1960s, in other regions such as Asia—including countries like India and Pakistan—the production of improved seeds promoted the cultivation of high-yield varieties of crops such as rice and wheat. However, this strategy of exploiting soil resources and the excessive use of agrochemicals led to the loss of agricultural biodiversity at both macro and micro levels, contamination of various aquatic and terrestrial systems, degradation and loss of soil health, and other negative effects on human and animal health [3].

At present, soil and plant-associated microbiota are considered part of the next Green Revolution because they emphasize sustainability as a key factor to avoid contaminating our soils and to keep them fertile and healthy [4]. Therefore, bioinoculants based on microbial agents, such as beneficial fungi and bacteria, can provide the same services as agrochemicals—whether promoting growth, improving nutrition, stimulating defense mechanisms against pathogen attacks, or, in general, increasing agricultural production. The global bioinoculants market is growing at approximately 10% annually and consists of billions of dollars in global sales [5], making it a tremendous opportunity to gradually phase out agrochemicals and increase agricultural production in a safe and healthy way for our ecosystems.

An alternative among the beneficial microbial communities in the soil is the plant growth-promoting rhizobacteria (PGPR). PGPR are part of the soil microbiome and fundamental to good soil health (among other physicochemical properties). Some well-known genera of PGPR include *Azospirillum*, *Arthrobacter*, *Bacillus, Pantoea*, and *Pseudomonas*, among others [6]. PGPR, through direct mechanisms, facilitate the acquisition of essential nutrients such as phosphorus, iron, and nitrogen, which are often scarcely available to plants due to their low solubility or chemical form in the soil [7]. For example, certain PGPR solubilize phosphorus by producing organic acids and enzymes that mineralize organic and inorganic forms of this element, improving its availability to plants. Additionally, bacteria such as *Pseudomonas* produce siderophores that sequester iron, a key nutrient for plant development, which can be absorbed by roots to promote growth. Regarding nitrogen, symbiotic bacteria of the genus *Rhizobium* and other nitrogen fixers convert atmospheric nitrogen into assimilable forms, increasing the nutrition of leguminous plants, while other PGPR stimulate this symbiosis. Also, the production of phytohormones is a direct growth-promoting pathway, including auxins, gibberellins, and volatile compounds that modulate physiological processes promoting germination, cell elongation, and root formation [8].

The indirect mechanisms through which plant growth-promoting bacteria (PGPB) benefit plants include antagonizing phytopathogens by restricting or eliminating their growth to protect plant health [9]. These bacteria produce compounds such as siderophores that sequester iron, limiting its availability to pathogens, and enzymes like chitinases, cellulases, and β-1,3-glucanases that degrade fungal cell walls. The genus *Bacillus* is especially notable for its biocontrol capacity, synthesizing a variety of ribosomal and non-ribosomal antibiotic peptides, as well as volatile compounds that induce defense responses in plants [10]. These responses increase resistance against pathogens, enhancing the protection and overall health of the crop.

The Bajío region of Mexico is considered one of the country’s main agricultural engines, characterized by fertile soils and a favorable climate that support the cultivation of maize, sorghum, wheat, strawberry, broccoli, chili, onion, and alfalfa [11,12]. The soils analyzed in this study are located within this region. Understanding the diversity of plant growth-promoting rhizobacteria (PGPR) in these soils and how plants recruit them through the root system is essential for harnessing their benefits in agriculture. Knowledge of microbial interactions and recruitment can guide the development of sustainable practices that reduce agrochemical use and improve soil health. Therefore, in this study, we analyzed bacterial diversity in two bulk clay soils with contrasting productivity levels and examined how this diversity shifts in the rhizosphere of maize (*Zea mays* L.) plants.

## 2. Results

### 2.1. Key Differences in Physicochemical Properties

The two soils analyzed were collected from agricultural fields in Salamanca and Villagrán, Guanajuato, Mexico, and are clay soils with similar physicochemical properties. The non-productive soil had a pH of 8.6, 2.4% organic matter, 38.6 ppm phosphorus, and 501 ppm potassium, whereas the productive soil showed a pH of 6.8, 5.22% organic matter, 38.9 ppm phosphorus, and 552 ppm potassium (Table 1). Most nutrients were comparable between soils, but manganese was higher in the productive soil (36 vs. 8.9 ppm), while sodium was lower (230 vs. 754 ppm).

As for the productive capacity of each soil, maize (*Zea mays* L.) and wheat (*Triticum aestivum* L.) yield records per hectare were evaluated over a five-year period from 2020 to 2024 (Table 2). These yields were consistently different and significant (Student’s *t*-test, *p* ≤ 0.05) across all the years analyzed, showing that the “productive” soil had a higher average yield than the “non-productive” soil for both maize (14,580 vs. 11,409 kg/ha) and wheat (7684 vs. 6562 kg/ha), respectively.

### 2.2. Evaluation of Bacterial Diversity Using 16S rRNA Sequencing

**Figure 1:**
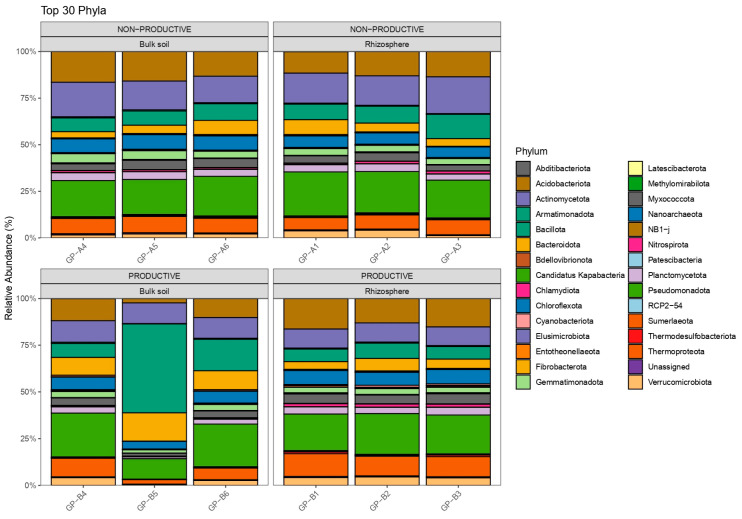
Taxonomic structure and relative abundances of the 30 most abundant phyla in bulk and rhizosphere samples from maize plants grown in non-productive and productive soils. GP samples represent three independent replicates per treatment.

**Figure 2:**
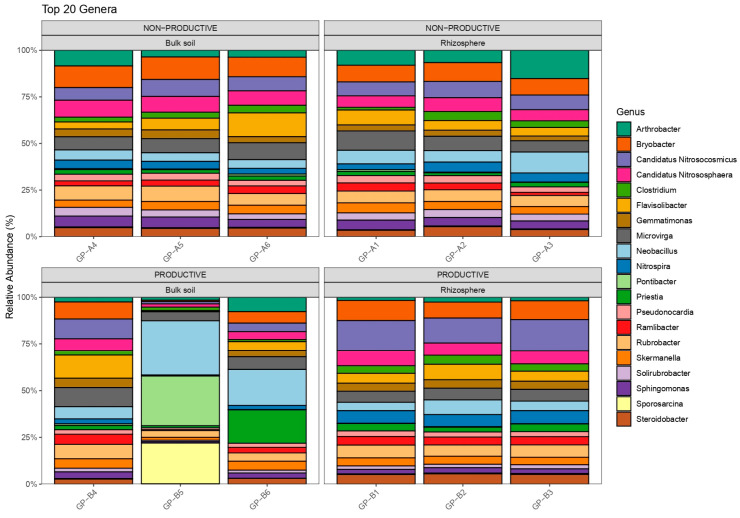
Taxonomic structure and relative abundances of the 30 most abundant genera in bulk and rhizosphere samples from maize plants grown in non-productive and productive soils. GP samples represent three independent replicates per treatment.

A total of 5,612,654 high-quality sequences were obtained after quality filtering, with an average of ~467,721 reads per sample. After denoising and chimera removal, 30,420 ASVs were retained. The ASV table was filtered to remove low-prevalence taxa and subsequently normalized to relative abundances for downstream analyses. Taxonomic classification of ASVs was performed using the SILVA v138.2 reference database. In both productive and non-productive soils, three ASVs were consistently among the most abundant prokaryotes, corresponding to the genera Candidatus *Nitrososphaera* and *Nitrososphaera* within the family Nitrososphaeraceae. This group of archaea, known as ammonia-oxidizing archaea (AOA), was detected in all samples from both soil types. Within the domain Bacteria, phylum-level diversity and relative abundance were very similar across the non-productive soil samples (Figure 1). In the productive bulk soil, however, one sample (GP-B5) displayed a markedly different profile, while the other samples showed broadly similar distributions (Figure 2). Genera such as *Arthrobacter*, *Neobacillus*, and *Microvirga* were among the most consistently detected in the bulk soil and rhizosphere of maize grown in non-productive soil. In the productive bulk soil, *Neobacillus* emerged as one of the most abundant genera, contrasting with its distribution in the non-productive soil. Other genera consistently observed in the bulk soil or rhizosphere of maize grown in productive soil included *Solirubrobacter*, *Flavisolibacter*, and *Priestia*, all of which are commonly associated with edaphic environments.

### 2.3. Alpha and Beta Diversity Analysis

**Figure 3:**
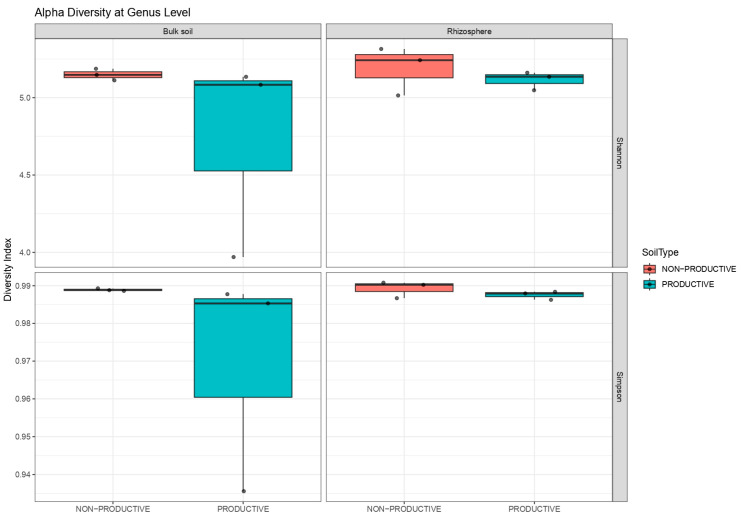
Shannon and Simpson ecological indices of alpha diversity in bulk soil and rhizosphere samples from non-productive and productive soils.

Alpha diversity indices revealed differences between soil types and compartments. In non-productive soils, Shannon and Simpson indices were similar between bulk soil and the rhizosphere, indicating comparable microbial diversity. In productive soils, bulk soil showed lower diversity values compared with the rhizosphere, as reflected by both Shannon and Simpson indices. Overall, rhizosphere samples tended to exhibit higher or more stable diversity than bulk soils, particularly in productive soils (Figure 3 and Supplementary Table S1).

**Figure 4:**
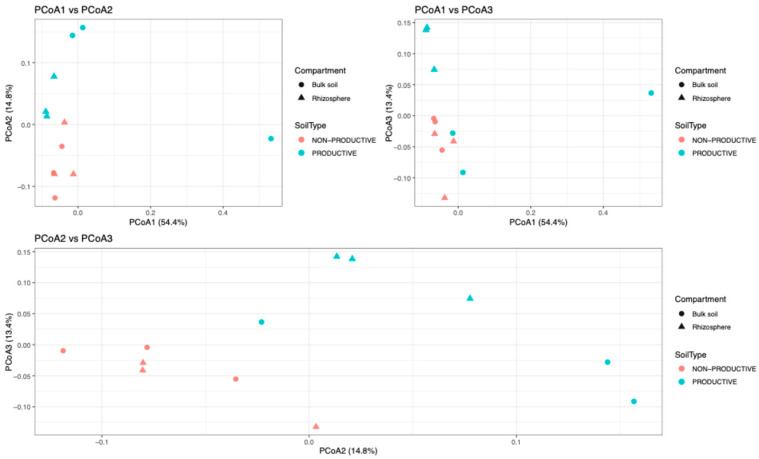
Comparison of prokaryotic community beta diversity among samples. Distances were visualized using principal coordinates analysis (PCoA).

Principal coordinates analysis (PCoA) of prokaryotic communities across soil samples revealed no clear clustering when grouped by compartment (bulk soil vs. rhizosphere) or soil type (productive vs. non-productive). The first three axes explain a substantial proportion of the total variance, with PCoA1 accounting for 54.4%, PCoA2 for 14.8%, and PCoA3 for 13.4%, resulting in a cumulative 82.6% of the variance explained by these axes. (Figure 4).

However, PERMANOVA based on Bray–Curtis distances indicated that the combined effect of soil type (productive vs. non-productive) and compartment (bulk soil vs. rhizosphere) significantly influenced microbial community structure (R^2^ = 0.284, *p* = 0.002). When evaluated separately, soil type explained a significant proportion of the variation (R^2^ = 0.177, *p* = 0.006), whereas compartment had a smaller and non-significant effect on community composition (R^2^ = 0.108, *p* = 0.216).

### 2.4. Differential Abundance and Key Shared Taxa

**Figure 5:**
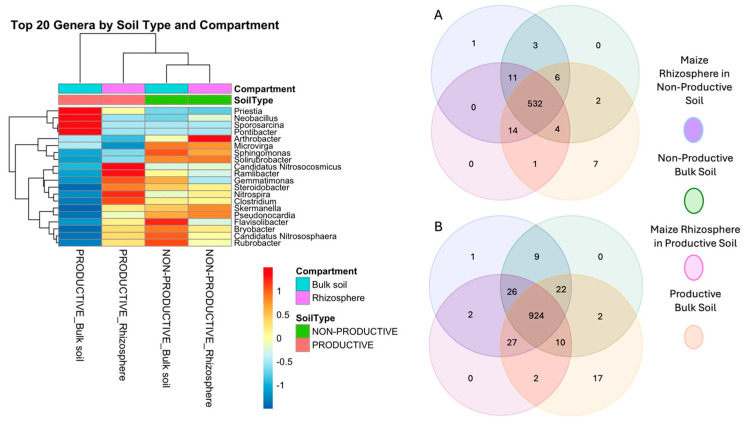
Heatmap of the top 20 prokaryotic genera by compartment (bulk soil and rhizosphere of maize plants) and soil type (non-productive and productive) shown in the left panel. The right panel shows a Venn diagram comparing unique and shared ASVs between the bulk soils and rhizospheres of maize plants grown in non-productive and productive soils.

Analysis of the 20 most abundant genera exhibiting the largest changes between compartments (bulk soil vs. rhizosphere) revealed a clear shift in rhizosphere communities. In non-productive soils, the rhizosphere showed reduced abundances of genera such as *Priestia*, *Neobacillus*, *Sporosarcina*, and *Pontibacter*, whereas these taxa remained largely unchanged in productive soils. In contrast, several genera, including *Candidatus*, *Ramlibacter*, and *Nitrospira*, increased markedly in the maize rhizosphere of productive soils. Additionally, *Flavisolibacter* decreased in abundance in the rhizosphere of non-productive soils, while *Arthrobacter* showed an increase (Figure 5, left panel).

**Figure 6:**
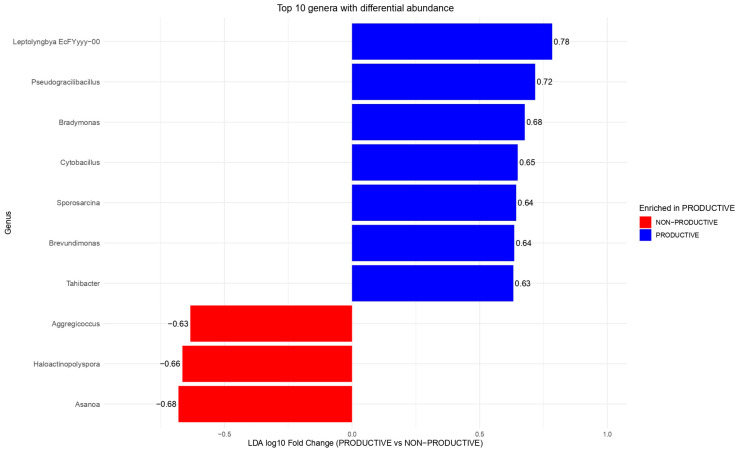
Linear discriminant analysis (LDA) of the top 10 genera with differential abundances in non-productive and productive soils.

**Figure 7:**
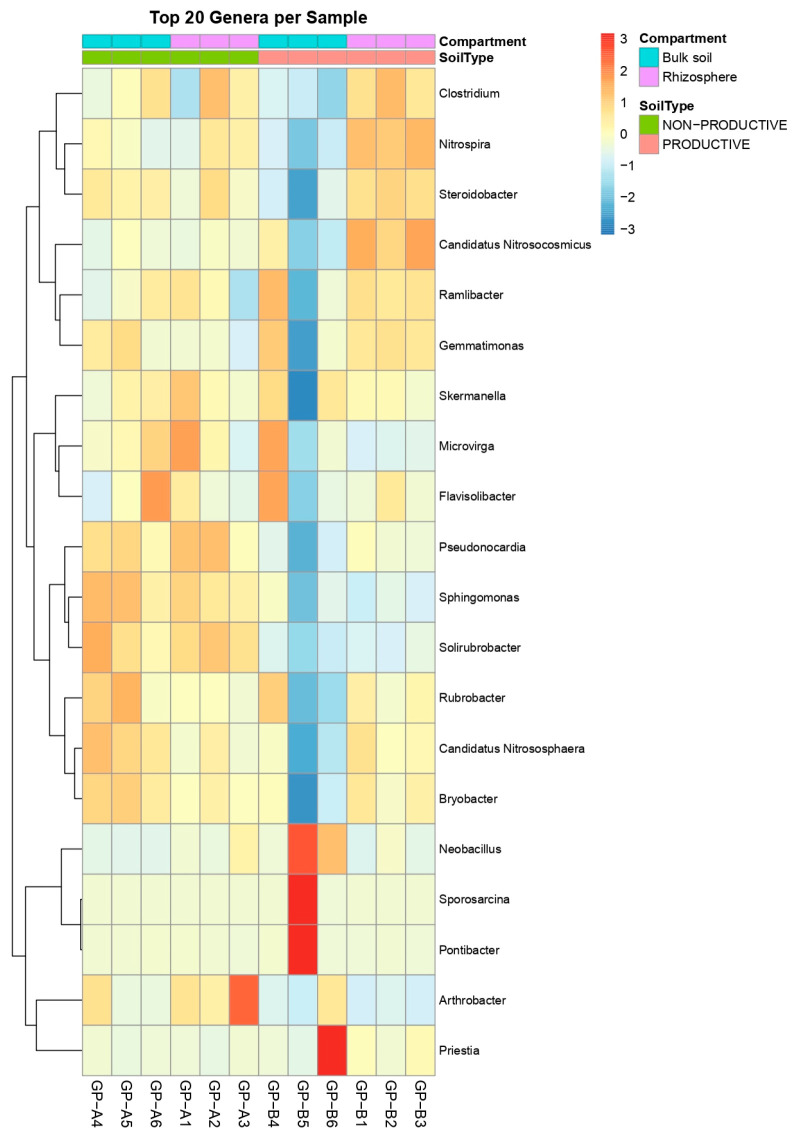
Heatmap of the top 20 prokaryotic genera per sample by compartment (bulk soil and rhizosphere of maize plants) and soil type (non-productive and productive).

Venn diagram analysis revealed a substantial core microbiota shared among all soil samples, comprising 532 genera (Figure 5, right panel, A) and 924 species (Figure 5, right panel, B). Consistent with this, LDA analysis at the genus level identified ten taxa with the greatest differential abundance, of which seven were enriched in productive soils, and three were reduced in non-productive soils (Figure 6). Together, these results indicate the presence of a broad core prokaryotic community shared between soil types, while also highlighting specific taxa whose relative abundance is differentially modulated in productive versus non-productive soils, with additional variability observed at the individual sample level (Figure 7).

### 2.5. Co-Occurrence Networks

**Figure 8:**
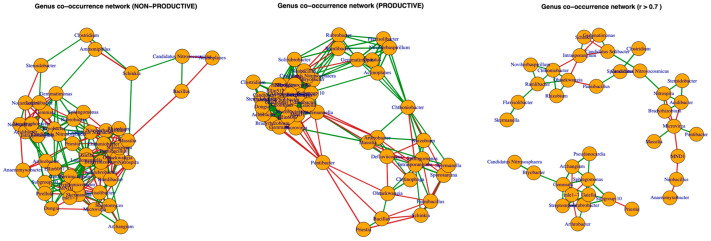
Co-occurrence networks of prokaryotic communities identified in non-productive (**left panel**), productive (**middle panel**), and combined (**right panel**) soil compartments. Negative interactions are shown in red, while positive interactions are marked in green. Correlation matrices (Spearman’s ρ) were calculated using relative abundances at the genus level, and only strong correlations (|ρ| > 0.7) were retained. Networks were constructed and visualized in R using the igraph package, which applies force-directed layouts; thus, node positions and distances have no intrinsic biological meaning and are shown solely to facilitate visualization of network topology.

Evaluation of genus-level interaction networks revealed distinct topologies in the non-productive soil, productive soil, and overall across both soils (Figure 8). In the non-productive soil, it is notable that positive interactions (shown in green) occur between Candidatus and *Bacillus* species, which are well-known as pathogen biocontrol agents and plant growth-promoting bacteria. However, negative interactions (shown in red) are also observed, for example, with *Massilia*.

Regarding positive interactions in the productive soil, it was found that *Flavisolibacter*, one of the most abundant genera, exhibits multiple positive interactions, whereas *Pontibacter* displays only negative interactions, even with plant-beneficial genera such as *Priestia* and *Bacillus*. In the combined analysis of both soils, the co-occurrence networks reveal that some nitrogen-fixing genera, including *Rhizobium* and *Bradyrhizobium*, form various positive interactions, while other genera, such as *Neobacillus* and *Priestia*, display negative relationships.

## 3. Discussion

This study characterizes prokaryotic communities in productive and non-productive soils across bulk soil and maize (*Zea mays* L.) rhizosphere compartments, revealing consistently high alpha diversity indicative of diverse and resilient soil microbiomes [13]. The similar diversity observed in the rhizosphere compared with bulk soil indicates that maize roots do not drastically reduce community richness, but may selectively modulate specific taxa. For example, the consistent detection of *Candidatus Nitrososphaera* and *Nitrososphaera* as abundant genera in all samples underscores the central role of ammonia-oxidizing archaea in both productive and non-productive soils [14]. These bacteria likely contribute to nitrogen cycling and suggest that key functional guilds are maintained regardless of soil productivity status. The ecological relevance of genes such as *amoA* within ammonia-oxidizing archaeal genomes, including *Nitrososphaera*, lies in their representation of the genetic potential for ammonium oxidation and nitrification [15,16]. Future studies will incorporate mRNA detection to assess the active involvement of this pathway under contrasting soil productivity conditions.

The higher productivity observed in the “productive soil” is likely associated with enhanced fertility and a microbial community enriched in taxa involved in nutrient cycling, particularly nitrogen transformations. The increased presence of nitrifying and plant growth-promoting microorganisms supports a close link between microbial functionality and soil fertility, reinforcing the role of microbially mediated nitrogen cycling in sustaining crop productivity [6]. As shown by Bei and colleagues [15] in an extensive meta-analysis of European agricultural soil microbiomes, members of the family *Nitrososphaeraceae* are both widespread and functionally relevant. In a previous study by the same research group, *Nitrososphaera* was identified among the five most abundant genera in loess chernozem-type soils of the Magdeburger Börde (Saxony-Anhalt, Germany), together with the detection of *amoA* genes encoding a subunit of the ammonia monooxygenase enzyme responsible for ammonia oxidation [16]. In addition, the predicted potential to produce phytohormone precursors suggests a plant growth-promoting (PGP) capability mediated by these metagenomically assembled genomes (MAGs). The authors also pointed out that soils from the German Magdeburger Börde region are well known for their high fertility [17].

In contrast, bacterial genera displayed different patterns depending on soil type and compartment. For instance, *Neobacillus*, *Priestia*, and *Flavisolibacter* were abundant in productive soils, consistent with their recognized roles in plant growth promotion and adaptation to edaphic environments, whereas *Arthrobacter* and *Microvirga* dominated in non-productive soils. These observations support the idea that soil physicochemical properties may shape bacterial composition more strongly than archaeal communities. The genera *Neobacillus*, *Priestia*, and *Flavisolibacter* have been widely reported as plant growth-promoting bacteria, including a strong association with maize crops. For example, ref. [18] Moturu reported the potential beneficial effects of *Priestia megaterium* and *Priestia aryabhattai* in maize crops, establishing associations with the plant as endophytes. Similarly, the newly described species *Neobacillus rhizosphaerae* has also been reported as a rhizobacterium associated with maize [19]. In the case of *Flavisolibacter*, it has been reported to be highly drought-tolerant and, due to its plant growth-promoting capabilities, to increase significantly in abundance in rhizospheric soils of maize crops inoculated with poultry manure [20].

Beta diversity analyses revealed that the compartment alone (bulk vs. rhizosphere) was not sufficient to generate distinct clustering, whereas soil type clearly defined microbial community structure. This finding underscores the influence of soil properties on rhizosphere effects in shaping overall community composition, particularly in soils with contrasting productivity [21]. Changes observed among the 20 most abundant genera (e.g., *Neobacillus*, *Priestia*, *Flavisolibacter*, *Candidatus Nitrososphaera*, and *Nitrososphaera*) between compartments—such as the increase in nitrogen-fixing taxa and archaea in the productive rhizosphere—highlight the selective enrichment of functionally important prokaryotes by maize roots, likely contributing to nutrient acquisition and plant health [22].

Regarding the genera most enriched in productive soils upon maize rhizosphere formation, *Leptolyngbya*, *Pseudogracilibacillus*, *Bradymonas*, and *Sporosarcina* (all members of the soil microbiome with diverse functional potentials) were notable [23,24], whereas Aggregicoccus, *Haloactinopolyspora*, and *Asanoa* decreased in abundance in non-productive soils. In the case of *Leptolyngbya*, it is capable of nitrogen fixation and biofilm formation, thereby promoting plant nutrition [25]; *Pseudogracilibacillus* contributes to phosphorus solubilization and is tolerant to water and salt stress [26]; *Sporosarcina* produces enzymes and bioactive compounds that may support plant growth and stress tolerance. Additionally, its role as an endophyte has been reported [27]. In contrast, the role of *Bradymonas* as a PGPR remains uncertain, and further investigation into its functions in the plant rhizosphere would be of interest. Co-occurrence network analyses revealed complex interaction patterns that varied between soils. Positive interactions between *Candidatus* and *Bacillus* in non-productive soils suggest possible synergistic effects on nutrient cycling and biocontrol, whereas negative interactions observed for *Pontibacter* in productive soils may indicate competitive exclusion or niche partitioning. *Bacillus* spp. are widely reported as plant growth-promoting bacteria and stimulators of soil health [28]. Similarly, in our research group, we have confirmed the beneficial function of several *Bacillus* strains (e.g., *B. velezensis* ITCE1) in promoting plant growth, as well as in biocontrol of phytopathogens that cause severe crop damage [29]. Interactions formed with other rhizosphere microbial groups, such as *Trichoderma*, have also revealed synergistic effects that benefit the plant [30]. In both soils, nitrogen-fixing genera formed multiple positive associations, emphasizing their ecological importance in maintaining soil fertility. These network patterns suggest that microbial interactions, rather than individual taxa alone, play a key role in modulating soil functionality and productivity [31].

Agronomic management was broadly similar across soils; however, the absence of quantitative data on agrochemical inputs represents a limitation, as it precludes a precise assessment of their contribution to the observed microbial patterns. Nevertheless, the consistent association of genera such as *Flavisolibacter* with soil productivity is supported by previous studies, including the work of Kruščić et al. [32], which identified this genus as responsive to biofertilizer and bacterial inoculant applications. These observations suggest that *Flavisolibacter* may represent a functionally relevant taxon linked to soil management and fertility-related processes.

Previous studies have established a strong link between soil microbial diversity and plant productivity; however, most have focused on single compartments or limited spatial scales [19,28]. In contrast, our study integrates field bulk soil diversity with maize (*Zea mays* L.) rhizosphere assembly across soil productivity gradients, thereby strengthening the functional link between microbial community structure and soil performance. In addition, our findings indicate the presence of a broad core microbiota shared between soils, along with key taxa whose abundance is differentially modulated by soil productivity and rhizosphere influence. This combination of stable core members and responsive taxa likely contributes to the resilience and functional stability of soil microbial communities [33,34]. From an applied perspective, the enrichment of beneficial genera, particularly nitrogen-fixing taxa, in productive soils highlights potential targets for microbial inoculants or soil management strategies aimed at enhancing plant growth and soil health.

## 4. Materials and Methods

### 4.1. Study Site and Sampling

**Figure 9:**
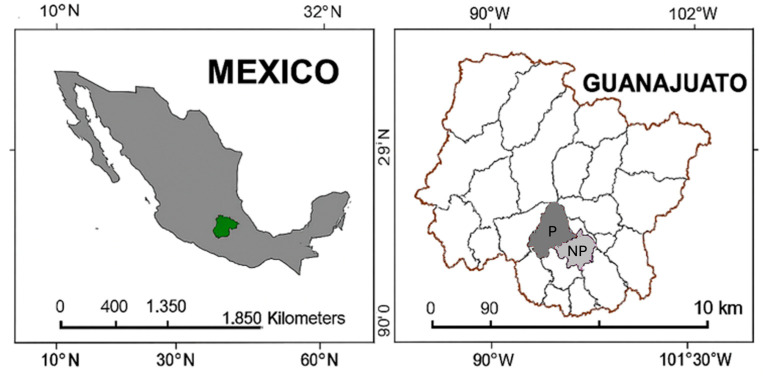
Sampling location coordinates in Guanajuato State, Mexico.

The soils sampled are located in the state of Guanajuato, specifically in the municipalities of Salamanca (20°35′53.2″ N, 101°12′03.2″ W) and Villagrán (20°30′22.9″ N, 101°02′50.7″ W), both under identical agronomic management but with significantly different levels of production. Salamanca soil is defined as a “productive soil”, while the Villagrán soil is designated as “non-productive” (Figure 9).

Samples were collected following a similar protocol for both soils. For rhizospheric soil, five maize plants were randomly selected from each treatment, and three independent replicates were generated per treatment. Roots were vigorously shaken to detach loosely adhering particles, and the soil tightly attached to the roots was collected as a composite sample of 10 g for metagenomic DNA extraction. For bulk soil, samples were taken at a depth of 10 cm from three different sites (with no plants around) within both productive and non-productive fields, pooled to obtain a composite sample of 10 g (in triplicate), using a sterile spatula, and transferred into sterile Falcon tubes. All samples were transported on ice to the laboratory for immediate processing.

### 4.2. DNA Extraction and Microbial Soil Sequencing

Metagenomic DNA was extracted from each composite sample using the DNeasy PowerSoil kit (QIAGEN, Düsseldorf, Germany), following the manufacturer’s instructions. To meet sequencing requirements, metagenomic DNA purity, integrity, and concentration were assessed using a Nanodrop 2000c spectrophotometer and agarose gel electrophoresis (1% agarose dissolved in 1X TAE). The hypervariable V3–V4 region of the 16S rRNA gene was sequenced. Sequencing was performed on an Illumina MiSeq platform (2 × 300 PE) using the sequencing services of MR DNA (Shallowater, TX, USA).

### 4.3. Sequence Processing and Quality Assurance

Raw paired-end reads were analyzed in R using the DADA2 package [35]. Sequence quality was initially evaluated using plotQualityProfile. Adapter sequences from the first 17 nucleotides were trimmed, and reads were reduced to 250 bp in length after quality assessment. Reads with ambiguous bases (N), an expected error value > 2, or mapping to the PhiX control sequence were removed.

### 4.4. Inference of Amplicon Sequence Variants (ASVs)

Reverse and forward read error models were calibrated independently and then used to dereplicate the sequences with DADA2’s core sample inference algorithm. The reverse and forward reads were merged to create full-length amplicons, which were tabulated in an amplicon sequence variant (ASV) format. Chimeras and duplicates were detected and excluded using the consensus method in DADA2.

### 4.5. Taxonomic Classification

Non-chimeric ASVs were assigned taxonomy using the SILVA reference database (v138.2). Taxonomic classification was output as .csv and .rds files, containing tables of ASV counts and taxonomy. Tables of summed counts were also generated at the phylum, genus, and species levels for downstream ecological analysis.

The ASV table, taxonomic classification, and metadata for compartment and soil type for each sample were included in a phyloseq object [36]. This object served as a template for ecological analyses, including diversity estimates, prevalence filtering, and community structure visualization.

To minimize noise from rare taxa, ASVs present in fewer than two samples were excluded. The remaining counts were converted to relative abundances (compositional transformation), allowing proportions to be directly compared across samples with different sequencing depths.

Alpha diversity was estimated using Shannon and Simpson indices. To mitigate uneven sequencing depth, diversity indices were computed on both raw and rarefied data. Group differences (e.g., Productive vs. Non-Productive soils) were tested with the Kruskal–Wallis test, adjusting *p*-values by the Benjamini–Hochberg method.

To compare microbial community composition among samples, PERMANOVA was performed based on Bray–Curtis distances, which were visualized using principal coordinates analysis (PCoA).

### 4.6. Community Structure and Visualization

The community structure of microbes was investigated across various levels of the taxonomic hierarchy. Phylum- and genus-level relative abundance stacked bar charts were planned, highlighting the 20 most dominant taxa, with remaining taxa grouped as “Others.” Heatmaps for the top 20 genera were generated by aggregating ASVs to the genus rank, scaling abundances row-wise, and stratifying by soil compartment and type.

### 4.7. Differential Abundance Analysis

Linear discriminant analysis (LDA) was used to identify the microbial genera that best discriminated between productive and non-productive soils. The analysis was performed on the ten genera exhibiting the highest differential relative abundances between soil types. Prior to LDA, relative abundance data were normalized to ensure comparability across samples. The LDA model was applied to maximize between-group variance while minimizing within-group variance, and discriminant scores were used to identify taxa driving soil-type separation. The top ten genera exhibiting the most pronounced differential abundance patterns were visualized using bar plots based on relative abundance.

### 4.8. Co-Occurrence Network Analysis

Co-occurrence networks were developed to investigate potential interactions among microbes. Correlation matrices (Spearman’s ρ) were calculated for relative abundances at the genus level. Positive and negative microbial interactions were inferred from Spearman rank correlation coefficients, where positive correlations (ρ > 0.7) were interpreted as potential co-occurrence or cooperative associations, and negative correlations (ρ < −0.7) as potential co-exclusion or competitive interactions. Edges with |ρ| > 0.7 were retained, resulting in genus–genus co-occurrence networks. Additionally, bipartite genus–sample incidence networks were created. Networks were constructed independently for productive and non-productive soil samples, enabling direct comparison of co-occurrence structures between environments.

## 5. Conclusions

This study indicates that differences in crop productivity are associated with shifts in specific microbial taxa and interaction networks rather than changes in overall alpha diversity. Compartment- and soil-type-dependent variations in key genera and their interactions suggest that a limited set of functionally relevant microorganisms may contribute disproportionately to soil productivity, highlighting potential targets for microbiome-based agricultural management.

## Figures and Tables

**Table 1:** Physicochemical properties of productive soil located in Salamanca and non-productive soil located in Villagrán, State of Guanajuato, Mexico.

Parameter	Unit	Salamanca (Productive Soil)	Villagrán (Non-Productive Soil)
Textural class	–	Clay	Clay
Saturation point	%	68	60
Field capacity	%	36.65	32.10
Permanent wilting point	%	21.70	19.10
Hydraulic conductivity	cm·h^−1^	0.60	0.28
Bulk density	g·cm^−3^	1.13	1.24
Organic matter	%	5.22	2.40
Phosphorus (P-Bray)	ppm	38.9	38.6
Potassium (K)	ppm	552	501
Calcium (Ca)	ppm	3892	4243
Magnesium (Mg)	ppm	858	532
Sodium (Na)	ppm	230	754
Iron (Fe)	ppm	12.7	8.15
Zinc (Zn)	ppm	0.56	0.32
Manganese (Mn)	ppm	36.1	8.90
Copper (Cu)	ppm	0.93	0.64
Boron (B)	ppm	0.69	2.57
Sulfur (S)	ppm	4.22	14.6
Nitrate (N-NO_3_)	ppm	14.9	5.36
pH (1:2 soil–water)	–	6.83	8.66
Total carbonates	%	0.01	1.70

**Table 2:** Grain yield (kg/ha) of maize and wheat cultivated in productive and non-productive soils during 2020–2024. Values represent means across replicates. Mean ± standard deviation; different letters indicate significant differences (*t*-test, *p* < 0.05).

Year	Maize—Productive Soil	Maize—Non-Productive Soil	Wheat—Productive Soil	Wheat—Non-Productive Soil
2020	16,360	13,280	7390	5690
2021	13,160	11,125	7840	6450
2022	14,590	12,135	7670	6590
2023	15,900	10,128	8050	7940
2024	12,890	10,380	7470	6140
Mean annual yield	14,580 ± 1564 a	11,410 ± 1305 b	7684 ± 270 a	6562 ± 844 b

## Data Availability

The datasets supporting the conclusions of this article are available in the National Center for Biotechnology Information (NCBI) repository under the accession number PRJNA1368882.

## Supplementary Materials

The following supporting information can be downloaded at: https://www.mdpi.com/article/10.3390/plants15010130/s1.
